# Development and validation of broad-spectrum magnetic particle labelling processes for cell therapy manufacturing

**DOI:** 10.1186/s13287-018-0968-0

**Published:** 2018-09-26

**Authors:** Richard Harrison, Hilda Anaid Lugo Leija, Stephanie Strohbuecker, James Crutchley, Sarah Marsh, Chris Denning, Alicia El Haj, Virginie Sottile

**Affiliations:** 1 Wolfson Centre for Stem Cells, Tissue Engineering and Modelling (STEM), School of Medicine, The University of Nottingham, Nottingham, NG7 2RD UK; 2 Institute for Science and Technology in Medicine-Keele University, Stoke-on-Trent, ST4 7QB UK

**Keywords:** Magnetic particles, Cell functionalisation, Cell therapy, Stem cells, Cell labelling

## Abstract

**Background:**

Stem cells are increasingly seen as a solution for many health challenges for an ageing population. However, their potential benefits in the clinic are currently curtailed by technical challenges such as high cell dose requirements and point of care delivery, which pose sourcing and logistics challenges. Cell manufacturing solutions are currently in development to address the supply issue, and ancillary technologies such as nanoparticle-based labelling are being developed to improve stem cell delivery and enable post-treatment follow-up.

**Methods:**

The application of magnetic particle (MP) labelling to potentially scalable cell manufacturing processes was investigated in a range of therapeutically relevant cells, including mesenchymal stromal cells (MSC), cardiomyocytes (CMC) and neural progenitor cells (ReN). The efficiency and the biological effect of particle labelling were analysed using fluorescent imaging and cellular assays.

**Results:**

Flow cytometry and fluorescent microscopy confirmed efficient labelling of monolayer cultures. Viability was shown to be retained post labelling for all three cell types. MSC and CMC demonstrated higher tolerance to MP doses up to 100× the standard concentration. This approach was also successful for MP labelling of suspension cultures, demonstrating efficient MP uptake within 3 h, while cell viability was unaffected by this suspension labelling process. Furthermore, a procedure to enable the storing of MP-labelled cell populations to facilitate cold chain transport to the site of clinical use was investigated. When MP-labelled cells were stored in hypothermic conditions using HypoThermosol solution for 24 h, cell viability and differentiation potential were retained post storage for ReN, MSC and beating CMC.

**Conclusions:**

Our results show that a generic MP labelling strategy was successfully developed for a range of clinically relevant cell populations, in both monolayer and suspension cultures. MP-labelled cell populations were able to undergo transient low-temperature storage whilst maintaining functional capacity in vitro. These results suggest that this MP labelling approach can be integrated into cell manufacturing and cold chain transport processes required for future cell therapy approaches.

**Electronic supplementary material:**

The online version of this article (10.1186/s13287-018-0968-0) contains supplementary material, which is available to authorized users.

## Background

Emerging cell-based therapies are demonstrating promising solutions to a wide range of debilitating health concerns facing global healthcare systems [1]. A number of stem cell sources are currently being researched for a range of potential applications yet challenges still exist, particularly with reproducible manufacturing, distribution [2, 3] and minimising costs of goods [4]. In addition to this, the mechanism of action underpinning many of these therapies remains largely unknown and subsequent cell dose requirements are high [5]. Better cell targeting strategies may help overcome some of these challenges, and magnetic particle (MP) labelling represents an attractive means to facilitate this targeting [6]. Magnetic targeting allows non-invasive imaging of administered cells post implantation [7], which can help measure efficacy and thus provide follow-up characterisation and safety data in the early stages of development [8]. Together, this spatial control over grafted cells and increased understanding with follow-up imaging have the potential to significantly improve the efficacy, specificity and impact of cell therapies for patient benefit [9].

MPs possess a wide variety of interesting and sometimes contrasting properties which make them useful for a multitude of biological and biomedical applications. With strong permanent magnets and superconducting electromagnets, a range of field strengths, gradients and durations are available for potential applications in multiple medical disciplines. The use of small magnetic particles for in-vitro diagnostics has been commonplace for almost 40 years [10–12]. The biomedical applications of magnetic particles have evolved over these years into a multidisciplinary field, which harnesses magnetic materials for a variety of applications. These multi-functional tools are often described as theranostics and describe the delivery of an active therapeutic payload to a cell whilst providing diagnostic information on its location [13].

The avenues that magnetic targeted therapeutics can take are diverse, including active material coatings [14], drug payloads [15] or targeted hyperthermia [16] to ablate carcinoma lesions at the cellular level. A multitude of research groups are working towards creating labelling solutions for stem cells, cell therapies and regenerative medicine products. The potential benefits of these products could include both magnetic tracking [17] and targeting [18] strategies, as well as sensory [19] and activation [20] particles.

MP cell tracking approaches are afforded through the use of magnetic resonance imaging (MRI). MP-based imaging stems from the first use of MRI contrast agents, which emerged around 1981–1982 [21] and allow identification of specific labelled areas within biological organisms. Recent advances in the MRI technique have enabled real-time qualitative analysis of in-vivo tissues providing real-time observations of disease progression or therapeutic efficacy [19, 22]. MRI is also of benefit to emerging targeting approaches using electromagnetic fields to guide magnetic particles. This technique utilises the magnetic scanner to guide magnetic objects and has been demonstrated to successfully deliver cell suspensions to the myocardium in animal models [23]. Permanent magnet-based approaches for magnetic cell temporal control are more common and have been demonstrated extensively in the wider literature. Magnetic induced migration of labelled cells towards specific areas in vivo has been shown for multiple tissue types, including the vasculature [24–30], retina [31, 32], CNS [33] and liver [34]. Finally, magnetically labelled cells have demonstrated utility in construction and handling of cell sheets for retinal applications [32] as well as engraftment into scaffold-like structures such as stents [35, 36]. This suggests some tissue engineering approaches may also benefit from labelled cell sources.

Despite the capability promised by developers of MPs to enhance the end regenerative medicine products [37], few of these products have been validated for clinical use [38]. Similarly, relatively little interest has been paid to how these MP theranostics could be incorporated into potentially scalable cell manufacturing platforms. Rather, existing published research focuses on labelling strategies in planar culture systems which would be unsuitable for wide-scale adoption [38]. Similarly, distribution of cell-based therapies has been under-studied and only recently is significant investment taking place in this space [2].

In order to produce the quantities of biological material for mainstream adoption of regenerative medicine and cell therapies, scale up production methodologies are needed. This should deliver any economies of scale and thus drive production costs down. We have previously described a safe and efficacious MP labelling strategy for human mesenchymal stromal cells (MSC) and demonstrated its utility as a multifunctional cell tracking and manipulation tool [38]. This paper describes labelling strategies using two MP size ranges (500 nm and 1000 nm) to label three therapeutically relevant stem cell populations: human bone marrow-derived MSC, iPS-derived human cardiomyocytes (CMC) and ReNeuron neural stem cells (ReN). We further describe how this can be integrated into the pooling or holding stage of a simulated cell manufacturing process and how the resultant cell product could be shipped at low temperature (4 °C) to a clinical environment in a ready-to-use state.

## Methods

All reagents were purchased from ThermoFisher Scientific (UK) unless otherwise stated.

### Human mesenchymal stromal cell culture

A human bone marrow-derived mesenchymal stromal cell line [38] (also known as mesenchymal stem cells) was cultured and expanded under standard cell culturing conditions (37 °C, 5% CO_2_) in standard culture medium consisting of Dulbecco’s modified Eagle’s medium (DMEM) supplemented with 10% (v/v) FBS, 1% (v/v) non-essential amino acids, 1 mM l-glutamine, 1 mM pyruvate and 1% penicillin/streptomycin. Cells were passaged using trypsin/EDTA. Primary mesenchymal stromal cells (pMSC) were isolated from human bone marrow aspirate (Lonza, UK) [39].

### Human neural stem cell culture

Immortalised ReNeuron VM cells (ReN) [40] were cultured on laminin-coated vessels in ReN NSC maintenance medium containing B27 neural cell supplement mix, bFGF (10 ng/ml) and EGF (20 ng/ml; Sigma Aldrich, UK). Cells were passaged every 3–4 days using trypsin (0.25%) and soybean trypsin inhibitor (Sigma Aldrich) solutions. Briefly, cells were rinsed with phosphate buffered saline (PBS) and then incubated in trypsin (0.25%) solution for 5–15 min until the cells detached. Twice the volume of soybean trypsin inhibitor (Sigma Aldrich) was added and the cells were centrifuged at 500 × *g* for 5 min. The cell pellet was re-suspended in fresh medium and plated in freshly laminin-coated flasks at a density of ~ 10,000 cells/cm^2^. All experiments presented in this study were carried out on cells between passages 5 and 8.

### Human iPS-derived cardiomyocyte cell culture

Cardiomyocytes (CMC) [41] were thawed, transferred to RPMI-B27 medium and centrifuged at 300 × *g* for 3 min. Cells were re-suspended in RPMI-B27 containing 10 μM ROCK inhibitor (Tocris Biotechne) and plated, with a first medium change carried out after 48 h with RPMI-B27 only. Subsequent medium changes were carried out thereafter at intervals of 2–3 days without ROCK inhibitor.

### Human cancer cell lines

HeLa (cervical cancer cell line) [42], HOS (human osteosarcoma cell line) [43], SHSY5Y (human neuroblastoma-derived cell line) [44] and Caco-2 (human epithelial colorectal adenocarcinoma cells) [45] were cultured and expanded in standard culture medium consisting of DMEM supplemented with 10% (v/v) FBS, 1% (v/v) non-essential amino acids, 1 mM l-glutamine, 1 mM pyruvate and 1% penicillin/streptomycin. Cells were passaged using trypsin/EDTA.

### Mouse embryonic stem cell culture

Mouse embryonic stem cells (ESC) were cultured in DMEM supplemented with 1.7 mM l-glutamine, 0.1 mM β-mercaptoethanol, 5 ng/ml mouse leukaemia inhibitory factor (LIF), 10% (v/v) FBS, 1% (v/v) non-essential amino acids, 1 mM pyruvate and 1% penicillin/streptomycin (stock 10,000 U/ml) without a feeder layer. Cells were dissociated by 0.05% trypsin/EDTA.

### Cell labelling with magnetic particles

Cells were seeded at 40% confluency and grown to 80% confluency before labelling. Fluorescently tagged magnetic particles of 500 nm and 1000 nm (ScreenMAG-Silanol, Chemicell, Germany) were used for cell labelling. Labelling of cell monolayers was performed as described previously [38, 46]. Briefly, adherent cell populations were incubated with MPs (10 μg Fe/ml standard dose or 25 μg Fe/ml for fully confluent cultures) in medium for 24 h. The next day, cells were thoroughly washed with PBS in order to remove excess particles attached to the cell surface or flask. For suspension cell labelling, MSC, CMC and ReN were evenly suspended in 7 ml growth medium without serum and MPs were added at 70 μg Fe of particles per 1 × 10^6^ cells. Cells were agitated at 60 RPM for 3 h and labelled suspensions were then centrifuged to remove excess particles before plating out or direct flow cytometry after fixation with 4% ice-cold paraformaldehyde (PFA) (VWR, UK).

### Particle labelling assessment

To measure particle uptake by flow cytometry, cells were harvested, centrifuged at 200 × *g* for 5 min and re-suspended in PBS prior to analysis. Fixed samples from suspension labelling were analysed in PBS immediately following PFA fixation. Labelled and unlabelled populations were compared to evaluate the percentage uptake based on fluorescent intensity. Analysis was performed on a Beckman Coulter FC500 8HT Flow Cytometer (Beckman Coulter, USA) with WEASEL (WEHI, Australia), using unlabelled cells as controls to evaluate increased fluorescence.

Particle uptake was further evaluated visually using fluorescence and super-resolution microscopy. Adherent cells from monolayer cultures or plated out after suspension culture were fixed with 4% PFA and stained using FITC-labelled Phalloidin (Life Technologies, USA) according to the manufacturer’s instructions [38, 47], following permeabilisation with 0.1% Triton X-100 for 5 min. Slides were incubated in a dark covered container at room temperature for 15 min, and then washed twice with PBS and counterstained with Hoechst 33342 (Sigma Aldrich, UK). Cells were then imaged using the Operetta High Content Analysis System (Perkin Elmer, USA). For super-resolution microscopy, CMC were seeded in Matrigel-coated glass-bottom culture dishes (MatTek Corporation, USA) and left to attach and beat for 3 days. Cells were then labelled with 10 μg Fe/ml for 24 h, washed three times with PBS and fixed with PFA.

### MSC osteogenic differentiation

MSC were seeded at 5 × 10^3^ cells/cm^2^ and the medium was then changed every 3 days for 14 days with either control medium or osteogenic induction medium containing DMEM supplemented with 100 nM dexamethasone, 0.05 mM l-ascorbic acid-2-phosphate and 10 mM β-glycerophosphate. Mineralised nodules were identified using Von Kossa staining [48]. Cells were fixed at room temperature for 15 min in 4% PFA, washed three times with dH_2_O and incubated with 1% silver nitrate in dH_2_O (Sigma Aldrich) under a UV lamp for 15 min. Samples were washed three times with dH_2_O, incubated for 5 min with 2.5% sodium thiosulfate solution (Sigma Aldrich), washed again with dH_2_O and imaged using an eclipse TS100 inverted microscope (Nikon, Japan).

### ReN differentiation

Cells were seeded at 10,000 cells/well onto laminin-coated 96-well plates (BD Biosciences) and expanded for 2 days in growth medium before initiating differentiation using ReN culture medium without growth factors [40]. After 7 days of differentiation, cells were fixed in 4% paraformaldehyde (PFA) for immunocytochemistry analysis.

### Cell viability assessment

Viability was evaluated using the resazurin metabolic assay, using a working solution consisting of 10% (v/v) Presto Blue stock solution prepared according to the manufacturer’s instructions. After 45-min incubation, the fluorescent signal of 100 μl samples was measured at 535 nm excitation and 615 nm emission in triplicate, using an Infinite 200 PRO plate reader and i-control software (Tecan, Switzerland). The positive control consisted of unlabelled cells which remained in monolayers for the duration of the labelling time period, and the negative control consisted of cells exposed to 70% methanol in H_2_O fixative for 10 min followed by 3× PBS washing.

### HypoThermosol refrigerated storage of cells

MSC, CMC and ReN at full confluency were removed from the incubator and the media exchanged with 4 °C HypoThermosol FRS preservation solution (BioLife Solutions, USA). Plates were then sealed with Parafilm (Bemis NA, USA) and stored at 4 °C for 24 h. Cells were then removed from refrigerated storage, and the HypoThermosol solution was replaced with warmed media. A recovery period of 48 h was then allowed prior to assessment or fixation with 4% PFA. Assessment of cells before and after HypoThermosol incubation was performed using bright-field microscopy. Images and videos were acquired using an eclipse TS100 inverted microscope (Nikon, Japan).

### Statistical analysis

Statistical analysis was in the form of ANOVA performed using GraphPad PRISM (GraphPad Software, USA). Significance was shown as *P ≤ 0.05, **P ≤ 0.01, ***P ≤ 0.001 and ****P ≤ 0.0001.

## Results

### Efficient cell labelling applicable to multiple cell types

**Fig. 1:**
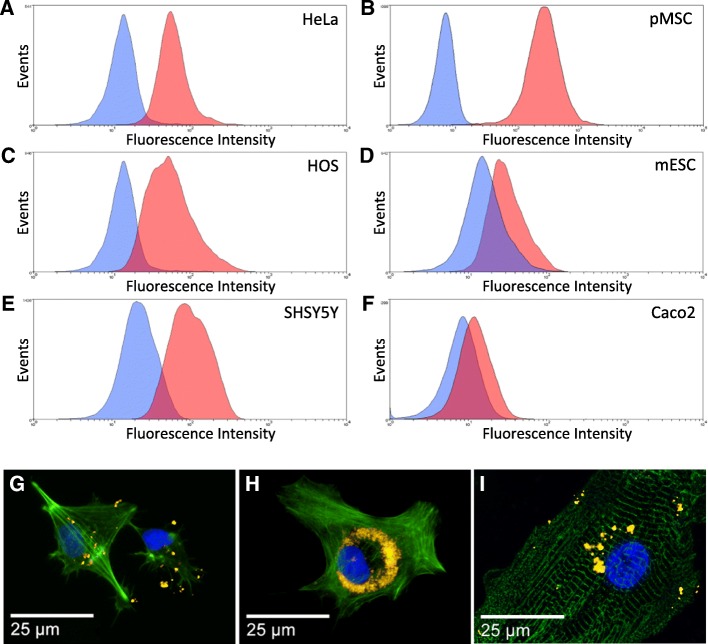
Microparticle-based labelling of adherent cultures incubated for 24 h in medium containing 1000 nm MPs (10 μg/μl). **a**–**f** Flow cytometry measurement of labelled (pink) and unlabelled (blue) cell populations analysed 24 h post incubation: **a** HeLa (cervical cancer stem cell line), **b** pMSC (primary mesenchymal stem cells), **c** HOS (human osteosarcoma stem cell line), **d** mESC (mouse embryonic stem cells), **e** SHSY5Y (human neuroblastoma derived stem cell line), **f** Caco-2 (human epithelial colorectal adenocarcinoma cells). Representative data shown, *n* = 3. **g**–**i** Fluorescence microscopy observation of neuroprogenitors (ReN, **g**), mesenchymal stem cells (MSC, **h**), and iPS-derived cardiomyocytes (CMC, **i**) incubated overnight with MPs (gold), stained with Phalloidin (green) and Hoechst 33342 (blue)

Therapeutically relevant cell types were cultured with fluorescently labelled MPs (10 μg/ml) in order to test the broad efficiency of the labelling strategy for regenerative medicine applications (Fig. 1). After 24 h of incubation, flow cytometry measurement showed labelling efficiencies ranging from above 23% (Caco-2 and mouse embryonic stem cells) to > 90% (HeLa, HOS, MSC and SHSY5Y) (Fig. 1a–f). Fluorescence imaging of three clinically relevant human cell types—cardiomyocytes derived from pluripotent embryonic stem cells (CMC), bone marrow-derived mesenchymal stromal cells (MSC) and neuroprogenitor cells (ReN)—incubated with fluorescent MPs using the same approach confirmed the intracellular labelling after 24 h (Fig. 1g–i).

**Fig. 2:**
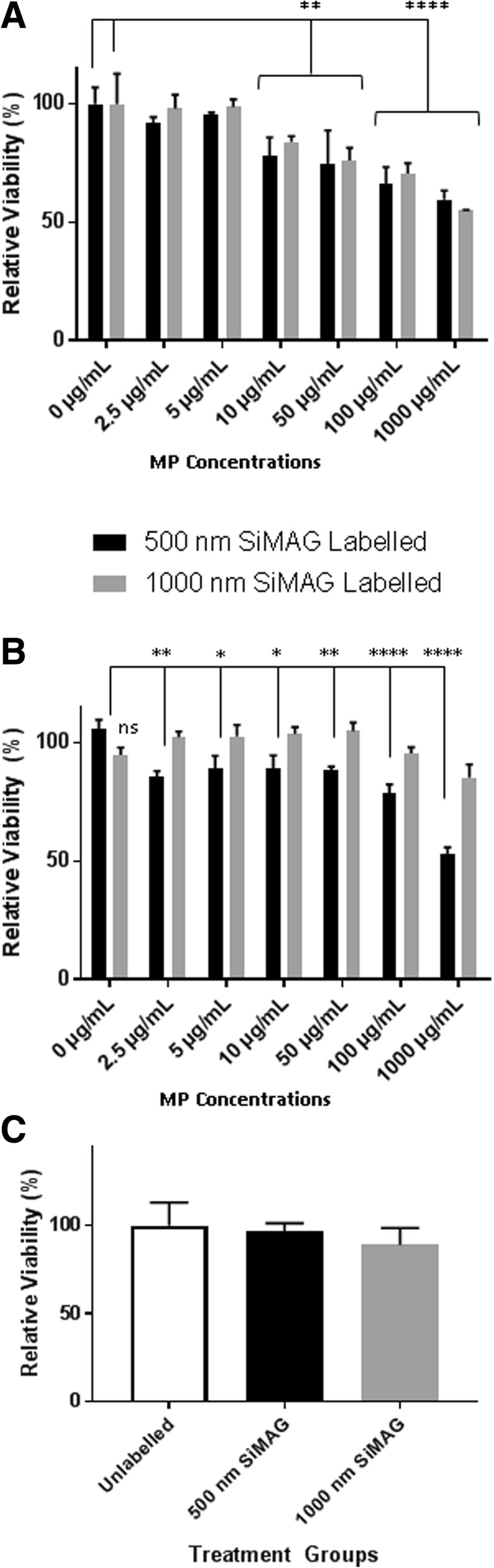
Metabolic activity measurements of MSC (**a**), CMC (**b**) and ReN (**c**) cultures at 24 h post labelling using 500 nm and 1000 nm MPs at a range of concentrations (**a**, **b**) including the standard 10 μg/ml dose (**c**). Error bars presented as SEM, *n* = 8 (**a**, **b**) and *n* = 3 (**c**). *P ≤ 0.05, **P ≤ 0.01, ****P ≤ 0.0001. MP magnetic particle

In order to assess the tolerance of these different cell types to the labelling procedure, metabolic activity was assessed at 24 h post labelling (Fig. 2), using 500 nm and 1000 nm MPs. No significant difference in relative viability was observed in MSC, ReN or CMC upon incubation with 500 nm or 1000 nm MPs after 24 h (Fig. 2a–c). At the standard 10 μg/μl concentration, cell viability was above 80%, indicating that magnetic labelling did not affect the cell cultures. Further viability, senescence and cell cycle profile assessments confirmed the maintenance of cellular integrity upon labelling (Additional file 1: Figure S1). Similarly, the unaffected morphology and viability of beating CMC were confirmed through observation of beating colonies (see methods in Additional file 2: Supplementary information, and videos of beating CMC colonies in Additional file 3: Figure S2 and Additional file 4: Figure S3).

### Cell labelling approach for suspension cultures

In order to assess the possibility of integrating MP labelling strategies within existing cell manufacturing platforms, labelling of cells was performed in suspension cultures. These corresponded to the cell pooling stages, where cells may be held in suspension between unit operations of a manufacturing process [49, 50]. This was evaluated for a range of cell types to show utility across a range of therapeutically relevant cells.

**Fig. 3:**
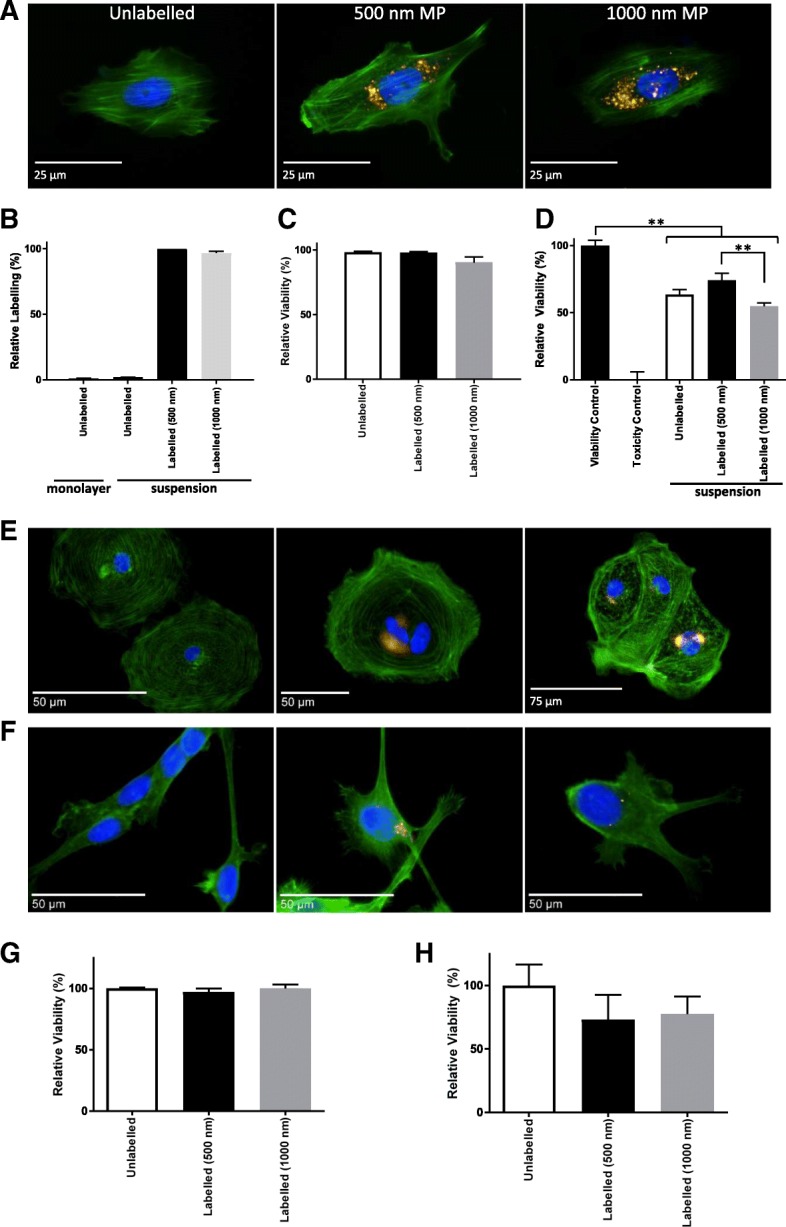
Development of suspension cell labelling approach for human cells. **a**, **b** MP-labelled MSC examined by fluorescence microscopy 24 h post labelling (**a**), after staining with Phalloidin (green) and Hoechst 33342 (blue), and by flow cytometry (**b**). **c**, **d** MSC viability examined by membrane integrity assay (**c**) and metabolic activity measurement (**d**) 24 h post labelling. Viable control consisted of unlabelled monolayer cells, Toxicity control consisted of cells exposed to 70% methanol for 10 min. **e**, **f** Fluorescence microscopy evaluation of CMC (**e**) and ReN (**f**) following suspension labelling with 500 nm (middle panel) or 1000 nm (right panel) MPs (gold) compared to unlabelled cells (left panel), with Phalloidin (green) and Hoechst 33342 (blue) counterstain. **g**, **h** Metabolic activity for CMC (**g**) and ReN (**h**) 24 h post suspension labelling. Error bars presented as SEM, *n* = 3. **P ≤ 0.01. MP magnetic particle

Suspensions of ReN, MSC and CMC were incubated with fluorescently tagged microparticles using two different sizes (500 nm and 1000 nm) (10 μg/ml) (Fig. 3). Successful labelling was confirmed with fluorescent microscopy for MSC (Fig. 3a), CMC (Fig. 3e) and ReN (Fig. 3f). The highest labelling levels were detected in MSC, as compared to CMC and ReN, although for the latter two the MP labelling was suboptimal for the imaging filters used in the microscope due to the maximum emission spectra not overlapping with the filters present. The labelling efficiency of MSC in suspension cultures was further examined by flow cytometry (Fig. 3b), which showed over 98% labelling of both MP types. Membrane integrity assays demonstrated no significant alterations in MSC populations after suspension labelling (Fig. 3c). Metabolic activity assayed 24 h after cell labelling indicated that MSC, CMC and ReN retained their relative viability following this suspension labelling procedure (Fig. 3d, g, h).

### Cold chain transport of therapeutically relevant labelled cells

**Fig. 4:**
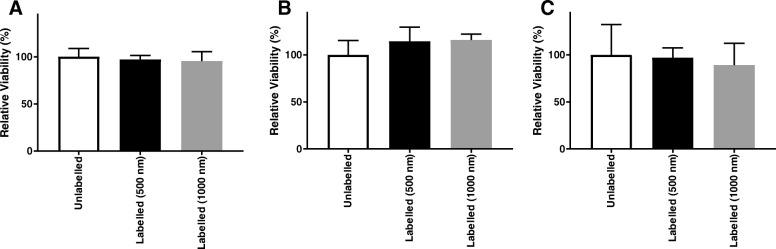
Metabolic activity measurements of MP-labelled MSC (**a**), CMC (**b**) and ReN (**c**) after 24 h in HypoThermosol conditions. Error bars presented as SEM, *n* = 6

The final component of the manufacturing value chain is transportation and distribution to the end user. This often overlooked step occurs outside the manufacturing facility and is a significant risk factor for introducing unknown changes to the final cell therapy product. The final stage transport of therapeutically relevant labelled cells was examined using HypoThermosol FRS as a transport medium in refrigerated environments. MSC, CMC and ReN were labelled with MP and stored in HypoThermosol preservation medium for 24 h at 4 °C to evaluate subsequent viability, functionality and label retention. Following a 24-h recovery period at 37 °C, cells were examined for metabolic activity as a measure of their relative viability (Fig. 4). No statistically significant decrease was detected in relation to the hypothermic incubation step, suggesting cells returned to basic metabolic activity post storage.

**Fig. 5:**
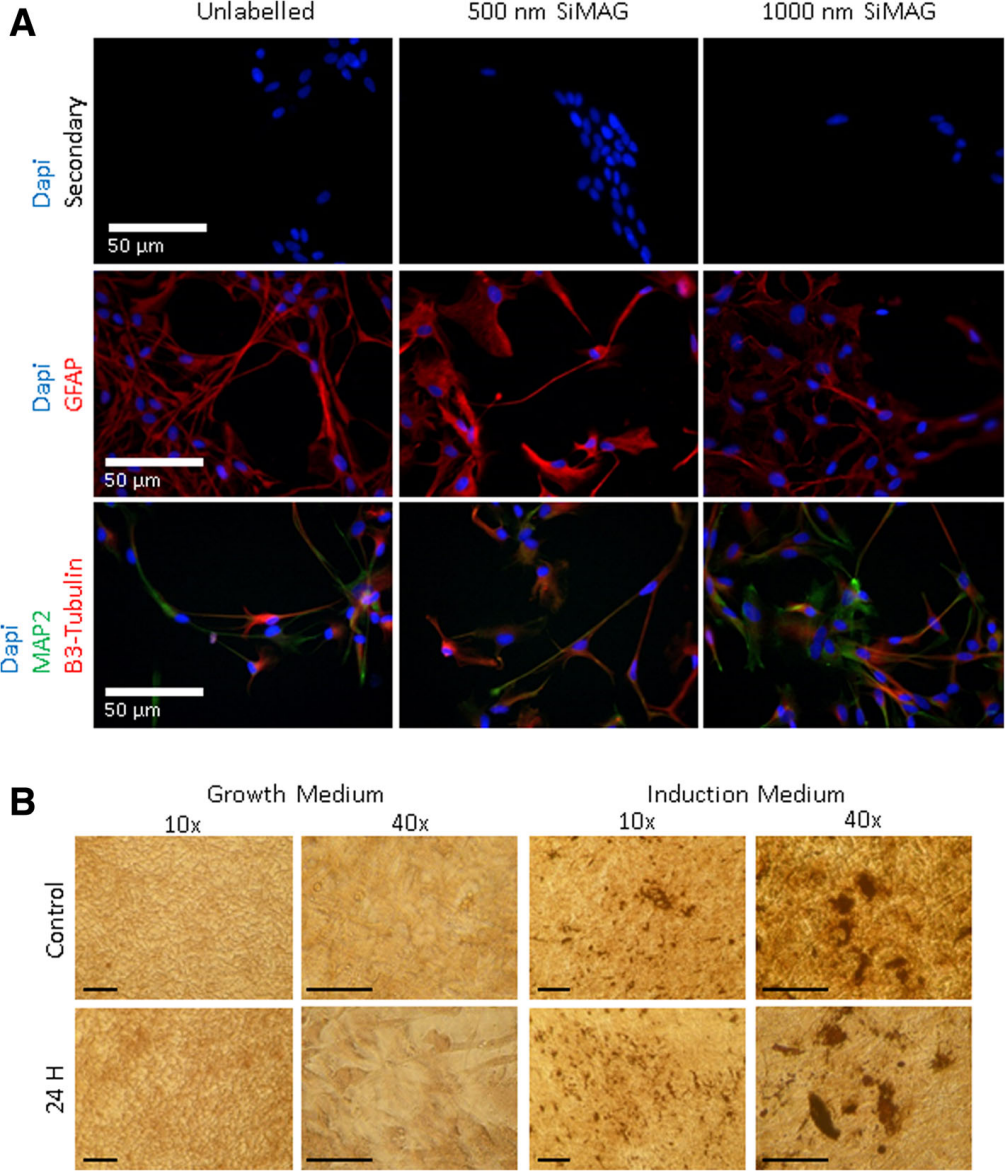
Assessment of labelled MSC and ReN after HypoThermosol storage for 24 h. **a** Differentiation of ReN analysed by immunodetection for MAP2, GFAP and β3-tubulin expression following a 7-day differentiation protocol. Additional examination of CMC forming beating cardiomyocyte clusters and analysis of alpha actinin expression in MP-labelled cell populations exposed to HypoThermosol storage shown in Additional file 5: Figure S4. **b** Osteogenic differentiation of MP-labelled MSCs for 14 days, post incubation in HypoThermosol analysed using Von Kossa staining to highlight mineral deposits formed after 14 days under induction treatment. Scale bar = 125 μm

It is crucial that cells remain not only viable but also functional. Previous work has examined tri-lineage differentiation of MSCs with and without iron MP labelling, demonstrating no impairment on the cells’ ability to differentiate [38, 46]. Thus, a simple indicator of functionality was examined for each cell type following the low-temperature storage (Fig. 5). MSC function was indicated using an osteogenic differentiation assay applied post 24-h storage. Von Kossa staining at day 14 highlighted mineralisation and the retention of their differentiation ability following labelling and storage of cells (Fig. 5a). Differentiation of the ReN carried out for 7 days post 24-h hypothermal storage indicated that both unlabelled and MP-labelled cells retained their ability to differentiate towards neuronal and glial lineages (Fig. 5b).

The morphology and functional ability of CMC to spontaneously beat was observed through bright-field imaging after 24 h (Additional file 5: Figure S4). This was further characterised through quantitation of alpha actinin immunodetection, which demonstrated expression in CMC following HypoThermosol incubation, albeit at a reduced level (Additional file 6: Figure S5).

## Discussion

Micron-sized iron oxide particles have demonstrated excellent utility for tracking by MRI, and early research suggests they have additional utility as theranostic agents [7, 51, 52]. Future cell therapies will require the adoption of scalable manufacturing processes enabling large-scale, reproducible manufacture [5, 53] that can be integrated into the manufacturing value chain rather than added post production, such as cell labelling [54].

Here, an approach to label a range of cell types efficiently with MPs was investigated. Labelling for 24 h was shown to be effective, and noted to be more efficient for larger cell types such as HeLa cells or MSC when compared to relatively smaller cells such as mouse embryonic stem cells or Caco-2 cells. When examining three therapeutically relevant human cell types, this was further confirmed as the smaller neuroprogenitor cells were observed to take up fewer particles than the larger CMC or MSC. Despite this, efficient MP labelling was demonstrated in monolayers for the MSC, CMC and ReN cell types. Whilst the relationship between particle size and particle uptake is known [55], previous literature has reported that biological indicators such as cell density and proliferative state can affect uptake [56]; however, the role of cell size is less clear and may involve indirect links between biological processes and the MP physicochemical properties [57]. The reduced MP uptake in ReN may be related to their reduced ability to store particles in a reduced cytoplasmic volume [58], as internalised MPs have been shown to accumulate into perinuclear intracellular vesicles [38].

The maintenance of cell integrity upon labelling was confirmed by metabolic activity assays, found in agreement with a lack of senescence or cell cycle perturbation responses. These data are also in line with a lack of detectable DNA damage upon labelling [38]. Whilst iron particles have been suggested to cause oxidative damage leading to senescence in MSC populations [59], the results presented here are in line with previous reports suggesting there is no significant adverse effect on cells [38, 60, 61]. Both MSC and CMC demonstrated tolerance of MPs up to 100× standard dose (1000 μg/ml) which then led to a 50% reduction in relative viability. In CMC, the 500 nm particles appeared to be more toxic than the 1000 nm particles. This could be linked to the increased surface area for smaller particles, previously hypothesised to lead to membrane wrapping effects [62, 63] and binding of a larger number of cell proteins [64]. ReN tolerated standard doses of MPs (10 μg/ml) with no reduction in viability; however, higher doses had a notable deleterious effect on cell attachment, preventing effective assessment. Overall, these findings are in line with the wider literature, reporting enhanced tolerance to MP labelling for MSC [38] and CMC [65]. Conversely, it has been suggested that neural stem cells are more susceptible to the iron contained in particles through an oxidative stress mechanism [65, 66]. Further assessments such as evaluation of senescence, apoptotic and autophagy responses could provide complementary parameters to assess and compare the finer cellular effects of high MP doses on different cell types.

CMC were further shown to maintain their functional capacity to beating following MP labelling, and cells were seen to change their morphology at doses exceeding 100 μg/ml but retained the ability to beat at even the highest dose (1000 μg/ml). Previous research on magnetic tissue engineering of cardiac sheets has investigated the effect that 10 nm magnetite particles may have on electrical connections between cells which may affect beating. This demonstrated no adverse effect [67], confirming our observations. Previous studies have suggested MSC function remains following MP labelling, with cells able to undergo multi-lineage differentiation into osteogenic, adipogenic and chondrogenic cell types [38]. This varies between studies, however, with some research groups demonstrating reduced markers of function including colony forming ability and differentiation potential [68]. This is likely due to the highly variable nature of MPs used throughout the field as well as a strong dose-responsive relationship between MPs and function [65]. Neural cells seem to have a reduced tolerance of labelling procedures, yet limited data suggest they do retain some function post labelling, with a study investigating MRI of transplanted labelled cells demonstrating substantial migration over time in vivo [69].

Efforts have been made to find clinically suitable labelling methods for iron oxide-based particles to ensure they effectively label the desired cell populations. For certain particles, which do not interact with cells, the use of a transfection agent to enhance labelling has proved successful [70]. Here we used an approach enabling the autonomous magnetic labelling of cells without the addition of other agents to the manufacturing process [38], and demonstrated its effectiveness in monolayers as well as suspension cultures for three human cell types. Suspension labelling demonstrated not only effectiveness but also retention of relative cell viability, with no statistically significant reduction when compared to unlabelled populations. This demonstration is of particular significance given the expected commercial significance of suspension culture methods such as stirred tank [50] or rocking WAVE® [71] bioreactors. These technologies represent an opportunity to significantly drive down the production cost of goods [72] and thus enhance market adoption of cell therapy [50], so the applicability of this labelling process represents an attractive route for integrating MPs with cell therapies.

When compared to monolayer MSC labelling, suspension cultures demonstrated a reduction in viability in all suspension populations, in the presence or absence of MPs, suggesting the cell pooling phase may require optimisation rather than the labelling process itself. Previous approaches have worked to bypass the requirement of adherent cell cultures for labelling success, instead opting for functionalised particle surfaces which drive targeted uptake through receptor mediated methods [73]. Both the 500 nm and the 1000 nm particles used here were shown to rapidly label adherent cultures and suspension culture. This suspension labelling methodology reduces the necessity for specific particle surface functionalisation in order to convey cell uptake traits. Rather, the particles are spontaneously taken up, which would allow functionalisation steps to be avoided.

In order to replicate the final step of a potential clinical therapy, the cell transport phase to the point of care, a low-temperature storage process was tested on MP-labelled cells using the HypoThermosol FRS product. This has previously been shown to maintain viability of a range of unlabelled cells such as pluripotent derived cardiac [74], mesenchymal [75] and neural [76] cell types. Here hypothermal exposure was tested on MP-labelled cells, and indicators of viability and function were examined.

MP-labelled neuroprogenitors were demonstrated to retain the ability to readily differentiate following simulated cold chain transport, as seen for unlabelled cells. This is in line with previous findings which demonstrated a retention of differentiation capacity following labelling [77]. Additionally, whilst both studies demonstrate retention of differentiation capacity, there are likely underlying long-term biological consequences. This is reflected in the observations during this research and previous literature [77] showing changes to cell morphology. This is potentially driven by the production of reactive oxygen species by the iron particles within the cells which are able to alter the internal regulatory mechanisms of neural stem cells [78].

Particle toxicity has previously been reported in neuronal and glial cell types [79]. In this study, MSC demonstrated retention of their ability to expand and form osteogenic cultures upon treatment. This is in line with previous published reports demonstrating retention of viability, colony forming ability and differentiation capacity of MSC populations following labelling with micron-sized iron oxide particles [80]. Additionally, the outcome of the osteogenic assay is in agreement with our previous observation that human MSC retain the ability to differentiate to all three lineages post labelling [38].

MP-labelled CMC were shown to remain functionally active following this hypothermal step, with cells resuming their beating activity when returned to normal culture conditions. When this was further examined with alpha actinin detection, it was noted that population purity had decreased, suggesting that storage may be affecting the mature CMC population to some degree. This contrasts with existing findings which reported CMC could tolerate similar hypothermal storage for up to 7 days [74], and suggests this slight decrease may be due to the presence of iron particles within the cells. Demonstrating the applicability of labelled cells for hypothermic storage is important in qualifying their use for this transport methodology. Hypothermic storage represents an attractive solution for cellular delivery as it removes any cryopreserved resuscitation step, thus simplifying the final formulation step at the clinical site [81].

## Conclusion

Future prospects for precision medicine and regenerative medicine through the use of MPs are promising, and require careful process validation to ensure clinical need is addressed in the early stage of manufacturing design. This study suggests MP labelling could be incorporated into a cell manufacturing process for a range of therapeutically relevant cell types. Furthermore, MP-labelled cell populations were shown to withstand low-temperature storage without significantly impacting indicators of cell function, suggesting the process would be compatible with cold chain transport. These results suggest that MP-based theranostics can be effectively integrated into cell manufacturing processes to support their use in future potential cell therapies.

## Additional files

Additional file 1: **Figure S1.** Maintenance of cell integrity after cell labelling with 1000 nm SiMAG for MSC. (**A**) Live/dead cell assay (live, green; dead, red) showing live cell fractions remaining above the toxicity threshold (dashed line). Scale bar: 100 μm. (**B**) Senescence assay (blue signal) showing no significant change upon labelling. Scale bar: 50 μm. (**C**) Cell cycle analysis showing no significant difference between unlabelled and labelled cell populations. **p < 0.01, ***p < 0.001 (TIF 445 kb)

Additional file 2: Supplementary materials and methods (DOCX 14 kb)

Additional file 3: **Figure S2.** Beating cardiomyocyte cell cluster showing particles distributed across its mass. Demonstration of viable beating cells with fluorescent particles allowing imaging and tracking of particles in real time. Cells stained with Calcein AM (green) and labelled with SiMAG (red) (WMV 6783 kb)

Additional file 4: **Figure S3.** Video of beating CMC colonies. Morphology and ability of cardiomyocytes to beat following labelling demonstrated that cells retain the ability to beat even at the highest doses (WMV 4259 kb)

Additional file 5: **Figure S4.** Cardiomyocytes labelled with SiMAG resuming beating following refrigerated storage. Following incubation of cardiomyocytes with HypoThermosol in refrigerated conditions, cells were assessed to determine whether they resumed beating. They were found to beat within 12 h of being removed from refrigerated storage even when labelled with SiMAG (WMV 7173 kb)

Additional file 6: **Figure S5.** Assessment of labelled CMC after HypoThermosol storage for 24 h. Image analysis performed examining alpha actinin expression in MP-labelled cell populations exposed to HypoThermosol storage. Error bars presented as SEM, *n* = 3 (TIF 25 kb)

## Abbreviations

CMC: Cardiomyocytes
iPS: Induced pluripotent stem cell
MP: Magnetic particle
MSC: Mesenchymal stem cells
ReN: Neural progenitor cells
