# Supplementary figures and images for: Development and validation of broad-spectrum magnetic particle labelling processes for cell therapy manufacturing
Source: Stem Cell Res Ther. 2018 Sep 26;9:248. doi: 10.1186/s13287-018-0968-0 (PMC6158868; doi:10.1186/s13287-018-0968-0)

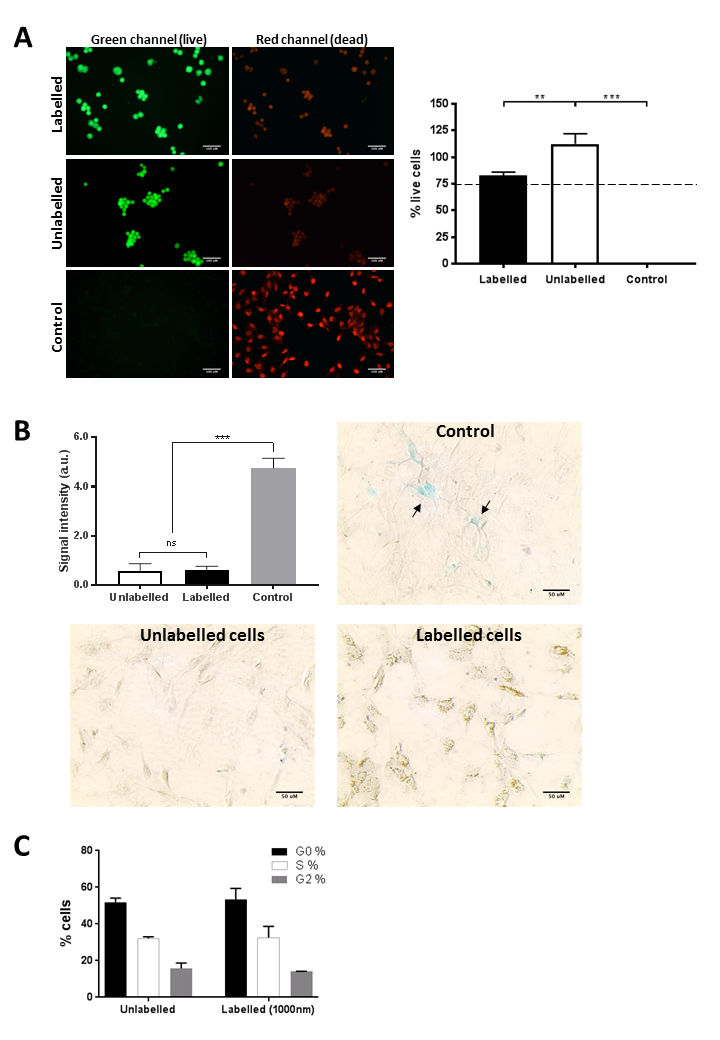

Supplement: Supplementary file 1 — Figure S1. Maintenance of cell integrity after cell labelling with 1000 nm SiMAG for MSC. (A) Live/dead cell assay (live, green; dead, red) showing live cell fractions remaining above the toxicity threshold (dashed line). Scale bar: 100 μm. (B) Senescence assay (blue signal) showing no significant change upon labelling. Scale bar: 50 μm. (C) Cell cycle analysis showing no significant difference between unlabelled and labelled cell populations. **p < 0.01, ***p < 0.001 (TIF 445 kb) [file 13287_2018_968_MOESM1_ESM.tif]

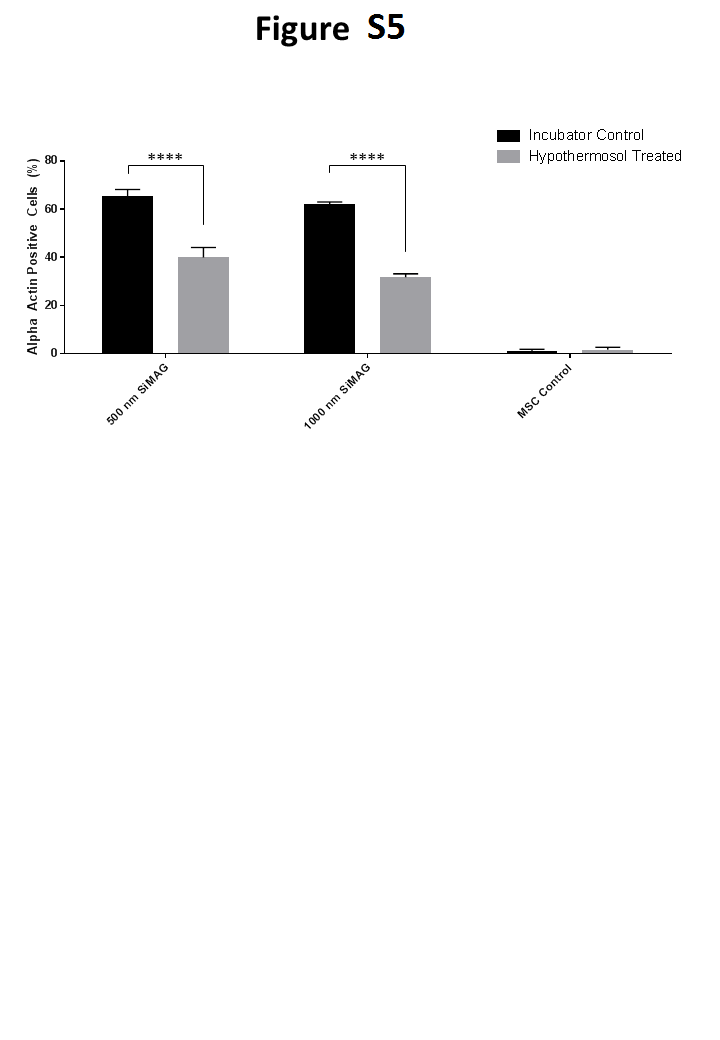

Supplement: Supplementary file 6 — Figure S5. Assessment of labelled CMC after HypoThermosol storage for 24 h. Image analysis performed examining alpha actinin expression in MP-labelled cell populations exposed to HypoThermosol storage. Error bars presented as SEM, n = 3 (TIF 25 kb) [file 13287_2018_968_MOESM6_ESM.tif]
