# Supplementary material for: Development and validation of broad-spectrum magnetic particle labelling processes for cell therapy manufacturing
Source: Stem Cell Res Ther. 2018 Sep 26;9:248. doi: 10.1186/s13287-018-0968-0 (PMC6158868; doi:10.1186/s13287-018-0968-0)
Supplement: Supplementary file 2 — Supplementary materials and methods (DOCX 14 kb) [file 13287_2018_968_MOESM2_ESM.docx]

**Supplementary Materials and Methods**

For cell viability, MSC labelled with 1000 nm SiMAG were stained cells using the Live/Dead® Viability/Citotoxicity kit (Molecular Probes, ThermoFisher Scientific) after 24 h. Cytotoxic controls were treated with 70% methanol for 30 minutes. Fluorescence was measured using a Tecan microplate reader (Ex/Em 495/530 – 528/645) and the percentage of viability was calculated according to manufacturer’s guidelines. Stained cells were imaged with a fluorescent Eclipse TS2 microscope (Nikon, Japan).

For senescence evaluation, MSC labelled with 1000 nm SiMAG (labelled) or unlabelled were fixed after 24 h cells and dyed using the Senescence β-galactosidase cell staining assay (Cell Signaling Technology) according to manufacturer’s instructions, imaged with an EVOS XI core microscope. Primary mouse MSCs were used as positive control. Three fields of view per condition were randomly selected at x100 magnification and staining signal was calculated using the image processing software ImageJ, by colour deconvolution using ROI from the positive images and substracting background signal from the particles.

For cell cycle analysis, cells were fixed after 24 h with 70% ethanol washed with PBS, and incubated 40 min with a 50 µg/mL propidium iodide (Sigma-Aldrich, UK) solution containing 0.1 mg/ml RNase A and 0.05% Triton X-100. Following two PBS washes, cell cycle analysis was performed on an FC500 Cytometer (Beckman Coulter, USA) using WEASEL (WEHI, Australia).

Functional Assessment of Cardiomyocytes: Beating cardiomyocytes were recorded following incubations using an Eclipse TS100 inverted microscope (Nikon, Japan). For CMC cells brightfield microscopy on a Leica IRE2 time-lapse DiC and fluorescence microscope with full stage incubation (Leica, Germany) was used to record videos of beating cell clusters. These were edited to identical timeframes for each cell concentration and assembled into a video to compare increasing doses of MPs.

For immunostaining, CMC were seeded at 5000 cells/well in Matrigel-coated 96 well plates and left to attach and beat for 3 days before labelling with 10 μg Fe/mL for 24 hours. Antibody detection of alpha actinin was performed using a monoclonal anti sarcomeric alpha actinin antibody (Sigma, A7811) with a secondary anti mouse alexa fluor 488 antibody. Imaging was performed on the Operetta High Content Analysis System (Perkin Elmer, USA).

For live fluorescent imaging of labelled cells, 500 nm SiMAG (red) labelled CMC were further stained with Calcein AM dye and imaged using a Cellavista microscope (Synentec, Germany).
